# Stereotactic biopsy of the breast using a decubitus table: comparison of histologic underestimation rates between 11- and 8-gauge vacuum-assisted breast biopsy

**DOI:** 10.1186/2193-1801-2-551

**Published:** 2013-10-22

**Authors:** Kyoung Eun Lee, Hak Hee Kim, Hee Jung Shin, Joo Hee Cha

**Affiliations:** Department of Radiology, Seoul Paik Hospital, Inje University, Seoul, 100-032 South Korea; Department of Radiology and Research Institute of Radiology, University of Ulsan College of Medicine, Asan Medical Center, 86 Asanbyeongwon-Gil, Seoul, Songpa-Gu 138-736 South Korea

**Keywords:** Stereotactic vacuum-assisted breast biopsy, Add-on unit, Decubitus table

## Abstract

**Purpose:**

To evaluate efficacy of the stereotactic vacuum-assisted breast biopsy(SVAB) using a decubitus table and to compare histologic underestimation rate between 11gauge(G)- and 8G-probes.

**Materials and methods:**

Pathologic results of SVAB using a decubitus table of 210(120 with 11G; 90 with 8G)-procedures in 208-women(median age, 48.8 years; range, 27-73 years) were retrospectively reviewed. SVAB was performed for suspicious microcalcification without mass on MMG and US. Surgury was performed for the diagnosis of malignant or high-risk lesion (65(31.0%)). Patients with benign diagnosis (120(57.1%)) underwent MMG follow-up (mean, 340-days). Histologic underestimation was defined as the need to upgrade ADH to DCIS or IDC, and DCIS to IDC at surgery. We analyzed the difference of procedure time, core number and core weight between 11G- and 8G-groups. Statistical significance was determined with chi-square test and 95%-CI for histologic underestimation, and student T-test for comparing two-groups.

**Results:**

Targeting was successful in all 210-biopsies on specimen radiographs. Mean core number, core weight and procedure time were 17.5 (17.5 ± 4.9), 1.57 g (1.57 ± 0.56), 34.5 min (34.5 ± 16.4) with 11G-probe, and 9.6 (9.6 ± 6.2), 1.83g (1.83 ± 0.93), 22.1 min (22.1 ± 12.5) with 8G-probe. Findings in 120 (57.1%) of the biopsies were benign, 36 (17.2%) were high-risk, and 54 (25.7%) were malignant. Two (6.25%) of 32 cases of ADH were upgraded to DCIS in 11G-group, and 2 (9.09%) of 22 in 8G-group. No case of DCIS was upgraded to IDC. There was no increase of complication in 8G-group than 11G-group.

**Conclusion:**

SVAB using a decubitus table is safe and effective method for the evaluation of suspicious microcalcification, and there was no significant difference between 11G- and 8G-probes. But, SVAB with 8G-probe is significantly more time efficient and effective procedure.

## Introduction

Stereotactic vacuum-assisted breast biopsy (SVAB) is well-established reliable, less invasive and cost effective method alternative to traditional needle localization biopsy (Sigal-Zafrani et al. 2008; Burbank 1997; Berg et al. 1997; Won et al. 1999; Peters et al. 2008; Zuiani et al. 2007; Kettritz et al. 2005; Diebold et al. 2005; Della Sala et al. 2004; Jackman 2004; Kettritz et al. 2004; Han et al. 2003; Rotter et al. 2003; Pandelidis et al. 2003; Liberman & Sama 2000). Stereotactic biopsy can be performed with dedicated prone system or add-on stereotactic unit (Georgian-Smith et al. 2002; Welle & Clark 1997; Sim & Kei 2008; Welle et al. 2000; Doyle et al. 1999). Dedicated prone systems are expensive and limited for one use, which guide needle and require significant space. Add-on units are less expensive and used with conventional mammographic unit. It can be used with the patient in the upright or lateral decubitus position. But the upright position is uncomfortable to the patient and causes higher frequency of vasovagal reaction. So, a technique using add-on stereotactic unit with the patient in the decubitus position is used increasingly to avoid patient movement and syncope (Welle et al. 2000; Doyle et al. 1999). Recently, the decubitus table (DBI™ table, Medical Positioning Inc, Washington, Kansas, U.S.A.) has been developed and used with add-on stereotactic unit.

Increasing needle diameter of SVAB allows larger samples of tissue to be obtained. Increasing sample weights has resulted significantly decreased rates of histologic upgrade between 11- and 14-guage needles (Darling et al. 2000). But larger biopsy needle has not decrease the upgrade rate of ADH between 9- and 11-gauge needles (Eby et al. 2009). However, SVAB using an 8-gauge needle has not been evaluated well on English literature.

So, the purpose of this study was to evaluate efficacy of the stereotactic vacuum-assisted breast biopsy (SVAB) procedure using a decubitus table and to compare procedure time, core number, weight, complication rate, and histologic underestimation rate between 11- and 8-gauge(G) probes.

## Materials and methods

We retrospectively reviewed data from 210 consecutive SVAB procedures in consecutive 208 women (median age, 49.8 years; range, 27-73 years) performed from June 2007 through May 2009. The study was approved by the institutional review board of Asan Medical Center.

All SVAB biopsies were performed with a decubitus table (DBI™ table, Medical Positioning Inc, Washington, Kansas, U.S.A.). Total 120 biopsies were performed with an 11-gauge probe (Mammotome, Biopsy/Ethicon endosurgery Inc, a Johnson & Johnson Co., Cincinnati, OH, U.S.A.). And total 90 biopsies were performed with an 8-gauge probe (Mammotome, Biopsy/Ethicon endosurgery Inc, a Johnson & Johnson Co., Cincinnati, OH, U.S.A.). MMG was taken with a Senographe DS with a stereotactic add-on unit (General Electric Medical System, Milwaukee, WI, U.S.A.).

The biopsy protocol and selection criteria for SVAB were identical for the 11G and 8G groups. Pre-biopsy medication restriction guidelines and routine post-biopsy care (manual compression, dressing, ice pack, and elastic band application) were also identical for these two groups. Stereotactic biopsy was used for calcifications without definite mass on MMG and no definite lesion on US. All biopsy targets were BI-RADS category 4 or 5 lesions and some 3 lesions which the patients want to biopsy. Lesion size and density of breast parenchyma were not used as criteria for exclusion from SVAB. Informed consent for biopsy was obtained from each patient. Images were obtained before and after the biopsy device were activated to document accurate needle position in the targeted lesion. Retargeting was performed if necessary. Biopsy specimens were typically obtained in a 360° rotation with the directional biopsy instrument, particularly when needle placement was within the lesion. When MMG obtained after the instrument was activated showed the needle to be immediately adjacent to the lesion, cores were obtained with the bowl of the needle directed toward the lesion. Standard practice at our institution at the time of the study was to obtain sufficient specimens to acquire adequate calcifications on routine specimen radiography. The radiologist assessed the specimen radiograph and obtained additional cores as needed. Post-biopsy MMGs were routinely obtained for the evaluation of residual calcification. Lesion type, number of cores obtained, and pathology results were recorded by the radiologist performing the procedure. Acute complications such as hematoma or bleeding also were recorded.

Surgical excision was subsequently carried out for the patients with the diagnosis of malignant or high-risk lesions on SVAB. Breast pathology was reviewed by two pathologists. The patients with benign diagnosis underwent MMG follow-up. The percentage of lesions diagnosed as ADH or DCIS at SVAB and the pathology result at surgery were compared between the 8G and 11G biopsy groups. Histologic underestimation was defined as the need to upgrade ADH to DCIS or IDC at surgery and to upgrade DCIS to IDC. And we analyzed the difference of total procedure time, core number and core weight between 11G and 8G groups.

The number of samples of 8G and 11G specimen was counted. The total weight of specimen was estimated by sample number × average weight per specimen (0.09 gram; 11G, 0.19 gram; 8G).

Histologic underestimation rate and complication rate for these two groups were analyzed for statistical significance by use of the chi-square test. Statistical significance was considered *p* < 0.05. The statistical calculations were performed with statistical software (SAS version 9.1.3, SAS Institute). The difference of total procedure time, and number of obtained samples were evaluated by the student T-test and 95% confidence interval.

## Results

The average of patient age was 48.8 years (range, 27–73 years) in the 11G group and 50.8 years (range, 30–72 years) in the 8G group. In the 11G group, 3 (2.50%) of the MMG findings were BIRADS 3, 113 (94.17%) were BIRADS 4, and 4 (3.33%) were BIRADS 5 lesions. In the 8G group, 2 (2.22%) of the MMG finding was BIRADS 3, 85 (94.44%) were BIRADS 4, and 3 (3.33%) were BIRADS 5 lesions. Sixty-five patients (31.0%) underwent surgical excision. MMG follow-up was available for 113 (53.8%) patients who did not undergo surgery (mean, 340 days). Remained 32 patients were followed up loss.

Targeting was successful in all 210 biopsies on specimen radiographs. A mean of 17.5 (17.5 ± 4.9) specimens per lesion were obtained with 11G probe and a mean of 9.6 (9.6 ± 6.2) specimens with 8G probe. The difference in number of specimens obtained per lesion between the 11G and 8G groups was analyzed for statistical significance with student T-test and was found statistically significant (*p* < 0.0001). Mean specimen weight was 1.57 g (1.57 ± 0.56) with 11G probe, and 1.83g (1.83 ± 0.93) with 8G probe. And mean procedure time of SVAB was 34.5 min (34.5 ± 16.4) with 11G probe, and 22.1 min (22.1 ± 12.5) with 8G probe. There were significant differences in procedure time (p < 0.0001), core number (p < 0.0001) and core weight (*p* < 0.0001).

Findings in 120 (57.1%) of the biopsies were benign, 36 (17.2%) were high-risk, and 54 (25.7%) were malignant on SVAB.

SVAB using decubitus table was tolerable in all patients and there was no vasovagal reaction or major complaint. Neither group had acute complications necessitating intervention. Two hematomas were reported in the 11G group and 1 in the 8G group, none of which required treatment. No infections were reported. And there was no statistically significant difference of complication rate in two groups (*P* = 0.71).

We reviewed the SVAB biopsy database to identify 61 (29.0%) patients in whom pathologic evaluation of stereotactic biopsy specimens yielded ADH or DCIS during the study period. Then, medical records were reviewed to determine the pathology result at final surgical excision. In 7 cases, surgical pathology reports were not available, usually because the patient did not want to undergo surgery or referred to other hospital. These cases were excluded from analysis. So, the remaining 54 cases were included in the evaluation of histologic underestimation rate.

On SVAB, 35 (29.17%) of 120 lesions were ADH or DCIS in 11G group, and 3 cases were excluded. In remaining 32 (26.7%) cases, 10 cases were ADH and 22 cases were DCIS. In 8G group, 26 (28.89%) of 90 lesions were ADH or DCIS, and 4 cases were excluded. In remaining 22 (24.4%) cases, 8 cases were ADH and 14 cases were DCIS.

**Figure 1:**
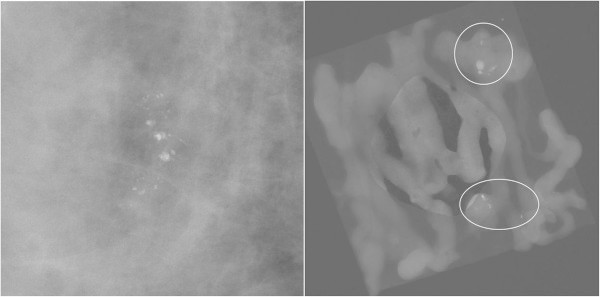
**The case of histologic underestimation on SVAB with an 11-gauge needle.** There are clustered coarse heterogeneous calcifications in the right breast which is BIRAD category 4. The pathologic result was mucocele-like lesion with ADH on SVAB, but mucocele-like tumor with DCIS on surgery.

**Figure 2:**
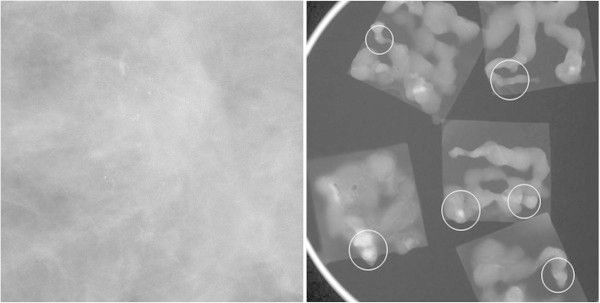
**The case of histologic underestimation on SVAB with an 8-gauge needle.** There are regional distributed amorphous calcifications in the left breast which is BIRAD category 4. The pathologic result was ADH on SVAB, but DCIS on surgery.

In the 11G group, 2 (6.25%) ADH lesions were upgrade to DCIS (Figure 1). In the 8G group, 2 (9.09%) cases were upgraded with one ADH lesion to DCIS and another DCIS lesion to IDC (Figure 2). The histologic underestimation rates between the 11G and 8G groups were not statistically different according to chi-square results (*p* = 0.706).

## Discussion

Stereotactic vacuum-assisted breast biopsy (SVAB) is used as an alternative method to the traditional needle-localization biopsy on recent clinical practice (Sigal-Zafrani et al. 2008; Burbank 1997; Berg et al. 1997; Won et al. 1999; Peters et al. 2008; Zuiani et al. 2007; Kettritz et al. 2005; Diebold et al. 2005; Della Sala et al. 2004; Jackman 2004; Kettritz et al. 2004; Han et al. 2003; Rotter et al. 2003; Pandelidis et al. 2003; Liberman & Sama 2000). It is well-known that SVAB is less invasive and cost effective method for the evaluation of suspicious microcalcification. And dedicated prone tables are well accepted for SVAB, but higher cost and require larger space, in addition to limited one use, which guide needle, prevent it to use widely. So, add-on stereotactic unit is developed. But vasovagal reaction is more common with the patient in the upright position. We used the decubitus table (DBI™ table, Medical Positioning Inc, Washington, Kansas, U.S.A.) which is recently developed for the use of SVAB with the patient lying in the decubitus position. Also, it has advantage that the patient movement is decreased due to comfortable position during procedure than with the patient in the upright position (Welle & Clark 1997; Welle et al. 2000; Doyle et al. 1999).

In our study, SVAB using a decubitus table was tolerable in all patients. The patients were comfortable during procedure, which had eliminated patient motion, so lesion targeting was successful in all SVAB. And there were no patients who complain of a vasovagal reaction and no significant immediate complication in this study.

Brem et al. (Brem et al. 2001) found that 8-gauge SVAB showed a 39% increase in sample weight compared with 11-gauge SVAB. We used estimated weights of tissue obtained with SVAB, which are 0.09 g with 11-gauge and 0.19 g with 8-gauge needle, to compare the obtained sample weight between two groups. Out result showed that obtained sample numbers in 8G group were significantly lower but sample weight were significantly heavier than those of 11G group.

Histologic underestimation remains an issue on SVAB in cases of ADH or DCIS, which are pathologic entities of having common features in some portion. Sampling error of SVAB can result histologic underestimation. Surgery is recommended for the diagnosis of ADH on SVAB to exclude the diagnosis of cancer. Darling et al. (Darling et al. 2000) reported that the frequency of histologic underestimation was substantially lower with 11G needle than with 14G needle. But, there are some reports that there were no significant differences of histologic underestimation rates between 11G and 9G SVAB (Eby et al. 2009; Brem et al. 2001; Lourenco et al. 2007). Brem et al. (Brem et al. 2001) showed the result that the accuracy of breast cancer diagnosis on SVAB with 8G needle was greater in lesions smaller than 30 mm compared with that with 11G needle. Our study showed similar result that there was no significant difference in histologic underestimation rate between 11G and 8G groups (*p* = 0.706).

Of the 156 benign and high-risk lesions, 113 lesions have been examined with MMG follow-up. All these lesions were stable during follow-up and none of these lesions have been subsequently proven to be malignancy.

Lomoschitz et al. (Lomoschitz et al. 2004) reported that histologic underestimation rate was not increased even if 12 samples rather than 20 samples per lesion had been retrieved and highest diagnostic yield was achieved at 11-gauge SVAB. Jackman et al. (Jackman et al. 2001) found that DCIS underestimation was 1.5 times more frequent with 10 or fewer specimens per lesion were obtained on SVAB using 11G and 14G needle. We found that histologic underestimation rate was not increased even if 9.6 specimens, fewer than 10 specimens per lesion, were obtained at SVAB using 8G needle compared with 11G needle. Specimen weight was 16.6% increased at 8G SVAB compared with 11G and the difference was statistically significant. But we suggest that it was not enough increase of specimen weight to decrease histologic underestimation rate. In our study, the determining factor of obtained specimen number was adequate retrieval of calcifications. It is more likely that more calcifications can be retrieved per specimen in 8-gauge SVAB, so the average number of specimen is smaller in 8G group than 11G group.

The limitation of our study include retrospective study design, which cause many follow-up loss, the difference of patient numbers between two groups - fewer 8G SVABs were included than 11G SVABs -, not all patients with ADH or DCIS on SVAB undergo surgery, and the amount of retrieved calcifications were determined by each radiologist who performed the SVAB procedure.

In conclusion, stereotactic vacuum-assisted breast biopsy using a decubitus table is safe and effective method for the evaluation of suspicious microcalcification. And there was no statistically significant difference between 11G and 8G needles in the diagnosis of suspicious microcalcification. But, SVAB using an 8G probe is significantly more time efficient and effective procedure than using an 11G needle. And further studies with larger patients and more long-term follow-up are needed to evaluate the efficacy of larger tissue obtaining device at percutaneous breast biopsy.

## Abbreviations

SVAB: Stereotactic vacuum-assisted breast biopsy
MMG: Mammography
US: Ultrasonography
ADH: Atypical ductal hyperplasia
DCIS: Ductal carcinoma in situ
IDC: Invasive ductal carcinoma.
